# Salicylic acid functionalized silica-coated magnetite nanoparticles for solid phase extraction and preconcentration of some heavy metal ions from various real samples

**DOI:** 10.1186/1752-153X-5-41

**Published:** 2011-07-15

**Authors:** M Reza Shishehbore, Abbas Afkhami, Hasan Bagheri

**Affiliations:** 1 Department of Chemistry, Yazd Branch, Islamic Azad University, Yazd, Iran; 2 Faculty of Chemistry, Bu-Ali Sina University, Hamedan, Iran; 3 Department of Chemistry, Takestan Branch, Islamic Azad University, Takestan, Iran

## Abstract

A method for the preconcentration of trace heavy metal ions in environmental samples has been reported. The presented method is based on the sorption of Cu(II), Cd(II), Ni(II) and Cr(III) ions with salicylic acid as respective chelate on silica-coated magnetite nanoparticles. Prepared adsorbent was characterized by XRD, SEM, BET and FT-IR measurements. The metals content of the sorbed complexes are eluted using 4.0 mL of 1.0 mol L^-1 ^nitric acid. The influences of the analytical parameters including pH, amount of solid phase and condition of eluting solution, the effects of matrix ions on the retention of the analytes were examined. The accuracy and precision of suggested method were tested by analyzing of certified reference materials. The detection limits (3S_b_/m, N = 8) for Cu(II), Cd(II), Ni(II) and Cr(III) ions are 0.22, 0.11, 0.27 and 0.15 μg L^-1^, respectively, and the maximum preconcentration factor is 200. The method was successfully applied to the evaluation of these trace and toxic metals in various waters, foods and other samples.

## 1. Background

Heavy metals are released into the environment from industrial applications, including mining, refining and production of textiles, paints and dyes. These pollutants greatly threaten the health of human populations and the natural ecosystems even at low concentration. As they do not degrade biologically like organic pollutants, their presence in drinking water or industrial effluents is a public health problem due to their absorption and therefore possible accumulation in organisms [1-5]. The toxicities of heavy metals may be caused by the inhibition and reduction of various enzymes, complexation with certain ligands of amino acids and substitution of essential metal ions from enzymes [4-6]. Hence, their determination in industrial effluents, various water resources, environmental and biological samples is important, especially in the environment monitoring and assessment of occupational and environmental exposure to toxic metals.

However, the direct determination of heavy metal ions at trace levels in real samples remains a challenging problem because of their low concentration and matrix effects even with frequently used sophisticated instrumental techniques such as inductively coupled plasma atomic emission spectrometry (ICP-AES), electrothermal atomic absorption spectrometry (ET-AAS) etc. without sample preconcentration and separation [7-10].

Flame atomic absorption spectrometry (F-AAS) is among the most widely used methods for the determination of the heavy metals at trace levels, but the sensitivity and selectivity of F-AAS is usually insufficient for the determination of heavy metals at trace concentrations in complex matrix environmental samples [11-13]. In trace analysis, therefore, preconcentration or separation of trace elements from the matrix is frequently necessary in order to improve their detection and selectivity by F-AAS [13-16]. Different techniques are used for the separation and preconcentration of metals in the solution. These include liquid-liquid extraction, precipitation, cation-exchange resins, cloud point extraction and solid phase extraction [2,17-20]. However, disadvantages such as significant chemical additives, solvent losses, complex equipments, large secondary wastes, prefiltration problems and time consuming procedures, limit the application of most of these techniques.

Solid phase extraction (SPE) addresses these problems. It can extend the detection limits and remove interfering constituents thereby improving the precision and accuracy of the analytical results. Activated carbon, polymeric fibers, Ambersorb, inorganic ion-exchanger, alumina and silica gel have been used to preconcentrate trace metal ions. However, they suffer from lack of selectivity, which leads to high interference of other existing species with the analyte metal ion and chemical stability [2,17].

Recently, using nanometer-sized materials in SPE as metals ions extractors has turned out to be an active area of research in the field of separation science because of their special properties. Magnetic nanoparticles, a new kind of nanometer-sized material, are widely used in the fields of biotechnology, biomedicine and as an efficient adsorbent with large specific surface area and small diffusion resistance [2,4,6,21-24]. The use of synthetic iron oxides is much more economical than commercial highly efficient activated carbon, in a 30:1 relative ratio depending on the particular kind of activated carbon [25]. The magnetic separation provides suitable route for online separation, where particles with affinity to target species are mixed with the heterogeneous solution. Upon mixing with the solution, the particles tag the target species. External magnetic fields are then applied to separate the tagged particles from the solution.

However, it should be pointed out that pure inorganic nanoparticles (such as Fe_3_O_4 _and Fe_2_O_3_) can easily form large aggregates, which may alter their magnetic properties [6,25]. Moreover, these nanometer-sized metal oxides are not target-selective and are unsuitable for samples with complicated matrices. Therefore, a suitable coating is essential to overcome such limitations. To overcome latter problem, chemical or physical modification of the sorbent surface with some organic compounds, especially chelating ones, is usually used to load the surface with some donor atoms such as oxygen, nitrogen, sulfur and phosphorus [2,6]. These donor atoms are capable of selective binding with certain metal ions. Salicylic acid (SA) is a commercial ligand with a carboxylic and a phenolic function site which can act as electron pair donors reacting with most of hard and intermediate cations. It has already been used, for example, as the modifier in chelating resins like Amberlite XAD-2-SA, Amberlite XAD-4-SA and silica gel-SA and it have shown good sorption capacity [26-28]. This may be due to the small size of ligand molecules that has facilitated extensive functionalization of the solid support matrices.

In this study, silica-coated magnetic nanoparticles modified with SA were synthesized by a sol-gel method. These magnetic nanoparticles were employed as an SPE adsorbent for separating and concentrating trace amounts of Cu(II), Cd(II), Ni(II) and Cr(III) ions from environmental and various other real matrices prior to their determination by F-AAS and was found to have superior preconcentration and metal loading ability compared to other adsorbents prepared using salicylic acid as the functional group. The propose method was validated by analyzing certified reference materials (both environmental and biological) and by performing recovery studies on water and food samples by F-AAS.

## 2. Experimental

### 2.1. Reagents and Apparatus

Chemicals used for experiments were all in analytical reagent grade. Aqueous solutions of chemicals were prepared with deionized water. The glass equipments kept in HNO_3 _10% (v/v) solution overnight and washed with deionized water several times, oven dried and kept in closed bags before use. Standard solutions of Cu(II), Cd(II), Ni(II) and Cr(III) ions were prepared from the nitrates of these elements each as 1000.0 mg L^-1^. Working solutions, as per the experimental requirements, were freshly prepared from the stock solution for each experimental run. pH adjustments were performed with 0.01-1.0 mol L^-1 ^HCl and NaOH solutions.

Certified reference materials such as vehicle exhaust particulates (NIES-8), human hair (NIES-5), tea leaves (NIES-7) and pepperbush (NIES-1) were obtained from the National Institute for Environment Studies (NIES). Zinc base die-casting alloy C (NBS-627) were provided by the Iron and Steel Institute of Japan (Tokyo, Japan) and the National Bureau of Standards, U.S. Department of Commerce, (Washington D.C., USA), respectively. A multivitamin capsule (bearing the commercial name Maxirich) was procured from Arjang pharmacy (Hamedan, Iran) and infant milk substitute, IMS, (commercially available as Lactogen 1), spinach, tomato and hydrogenated oil were obtained from the local market, Hamedan.

The concentration of metals was determined by atomic absorption spectrometry using a Varian model Spect AA 220 apparatus. The instrumental settings of the manufacturer were followed. Infrared spectra were recorded with a Fourier transform infrared spectrometer (FT-IR, Perkin Elmer, spectrum 100). Samples were gently ground and diluted in nonabsorbent KBr matrices to identify the functional groups and chemical bonding of the coated materials. Scanning electron microscopy (SEM) was performed to measure the particle size and shape (SEM-EDX, XL30 and Philips Netherland). The crystal structure of synthesized materials was determined by an X-ray diffractometer (XRD) (38066 Riva, d/G.Via M. Misone, 11/D (TN) Italy) at ambient temperature. Surface area and porosity were defined by N_2 _adsorption-desorption porosimetry (77 K) using a porosimeter (Bel Japan, Inc.). A Metrohm model 713 (Herisau, Switzerland) pH-meter with a combined glass electrode was used for pH measurements.

### 2.2. Preparation of samples

#### 2.2.1. Natural and sewage water samples

The water samples, river water (collected from Alvand, Hamedan, Iran), canal water (collected from Yazd, Iran), sewage water (collected from area in the vicinity of local nickel electroplating industry, Hamedan) and tap water (collected from our faculty) were immediately filtered through Millipore cellulose membrane filter (0.45 μm pore size), acidified to pH 2 ± 0.01 with HNO_3_, and stored in precleaned polyethylene bottles. After then, pH of the sample was adjusted to 6.0 and the procedure described in section 2.5 has been carried out.

#### 2.2.2. Digestion of standard environmental, biological and metal alloy samples

Two certified reference materials (CRMs); vehicle exhaust particulates (NIES-8), pepperbush (NIES-1) and tea leaves (NIES-7), were analyzed. Approximately 0.50 g of this material, were weighed accurately into a Teflon cup, and dissolved in concentrated nitric acid (~10 mL), with heating in a water bath. The solution was cooled, diluted and filtered. The filtrate was made to 100.0 mL, with deionized water in a calibrated flask. An aliquot of the sample solution was taken, and the target metals ions were determined by the given procedure.

The sample solutions of biological CRMs such as human hair (NIES-5) was prepared as proposed by International Atomic Energy Agency [29]. A 50.0 mg of each of the samples was agitated with 25 mL of acetone, and then washed three times with deionized water and with 25 mL of acetone. The washed samples were placed in a glass beaker individually and allowed to dry at room temperature. Decomposition of organic matter is an important part for determination of heavy metals in these samples. Therefore, each of the samples was dissolved in 10 mL of concentrated nitric acid. After adding 2.5 mL of 30% H_2_O_2 _the solution was boiled to dryness. The residue obtained was dissolved in minimum amount of 2% HCl and made up to a 50 mL volume in a calibrated flask. Then the procedure given in Section 2.5 was performed.

To dissolve the standard reference alloy, zinc based die-casting alloy C (NBS-627), 25 mg of the sample was taken into a beaker and dissolved in 10-50 mL of HCl:HNO_3 _mixture (3:1). The solution was boiled to near dryness. Finally, the residue was dissolved in minimum volume of 2% HCl and filtered through Whatman filter paper No. 1. The residue was washed with two 5 mL portions of hot 2% HCl. The solution was evaporated to dryness. The residue was dissolved in 5 mL of 2% HCl and make up to 50 mL with deionized water after its pH was adjusted to desired value.

#### 2.2.3. Preparation of multivitamin capsule and food samples

Five multivitamin capsules (5.83 g) were taken in a beaker containing 25 mL of concentrated HNO_3 _and digested by slowly increasing the temperature of the mixture to 40 ± 0.2°C. The solution was gently evaporated on a steam bath until a residue was left. It was subsequently mixed with 50 mL of deionized water and HNO_3 _was then added drop wise until a clear solution was obtained on gentle heating.

Powdered IMS food sample (200.0 mg) was heated in a beaker containing mixture of concentrated H_2_SO_4 _(20 mL) and HNO_3 _(10 mL) till a clear solution was obtained. It was allowed to cool and most of the acid was neutralized with NaOH. The total volume was made up to 50 mL with deionized water and kept as stock.

Hydrogenated oil (2.00 g) was taken in a beaker and dissolved in 15 mL of concentrated nitric acid with heating. The solution was cooled, diluted and filtered. The filtrate was made up to 50 mL with deionized water after adjusting its pH to the optimum value.

A 10 g sample of tomato sample was heated in silica crucible for 3 h on a hot plate and the charred material was transferred to a furnace for overnight heating at 650°C. The residue was cooled, treated with 10.0 mL concentrated nitric acid and 3 mL 30% H_2_O_2 _and again kept in a furnace for 2 h. The final residue was treated with 3 mL concentrated hydrochloric acid and 2-4 mL of 70% perchloric acid and evaporated to fumes. The solid residue was dissolved in water, filtered and the pH was adjusted to 6.0 by the addition of NaOH and HCl solutions. The preconcentration procedure given above in section 2.5 was then applied to these solutions.

0.1 g of vegetable sample was placed in a 100 mL beaker and 10 mL of concentrated HNO_3 _was added. The mixture was evaporated near to dryness on a hot plate at about 150°C. After cooling to room temperature, 3 mL of concentrated hydrogen peroxide was added. The mixture was again evaporated to dryness and the residue dissolved with 0.5 mol L^-1 ^HNO_3_. It was filtered through a filter paper. The preconcentration procedure was applied to this sample solution.

### 2.3. Preparation of silica-coated magnetite nanoparticles

The magnetite nanoparticles (MNPs) were prepared by the conventional co-precipitation method with minor modifications [30]. In this method, ultrasonic vibration by an ultrasonic bath was used instead of magnetic stirring. FeCl_3_.6H_2_O (11.68 g) and FeCl_2_.4H_2_O (4.30 g) were dissolved in 200 mL deionized water under nitrogen gas in an ultrasonic bath at 85°C for a few minutes leading to smaller and more homogenized particles. Then, 20 mL of 30% NH_3 __2_O, which is different from the 15 mL of 20% NH_3 __2_O used in Ref. [30], were added to the solution. The color of bulk solution changed from orange to black immediately. The magnetite precipitates were washed twice with deionized water and once with 0.02 mol L^-1 ^sodium chloride. The washed magnetite was stored in deionized water at a concentration of 40.0 g L^-1^.

Then, the magnetite suspension prepared above (20 mL) was placed in a 250 mL round-bottom flask and allowed to settle. The supernatant was removed, and an aqueous solution of tetraethoxysilane [TEOS, 10% (v/v), 80 mL] was added, followed by glycerol (60 mL). The pH of the suspension was adjusted to 4.6 using glacial acetic acid, and the mixture was then stirred and heated at 90°C for 2 h under a nitrogen atmosphere. After cooling to room temperature, the suspension was washed sequentially with deionized water (3 × 500 mL), methanol (3 × 500 mL), and deionized water (5 × 500 mL). The silica magnetite composite was stored in deionized water at a concentration of 40.0 g L^-1^.

### 2.4. Preparation of silica-coated magnetite nanoparticles modified with salicylic acid

**Figure 1:**
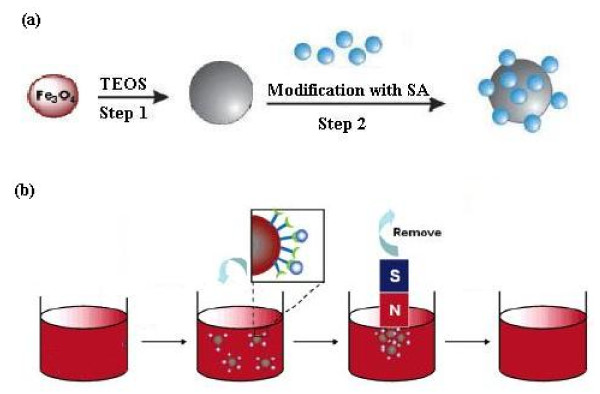
**Schematic of the preparation of adsorbent (a) and solid phase extraction of the analytes (b)**.

25 mL of silica-coated magnetite prepared as described above was washed with ethanol (2 × 100 mL) and then diluted to 150 mL with 3.3% SA solution and 16 mmol L^-1 ^acetic acid solution (pH 4.5). The solution was transferred to a 500 mL 3- necked round-bottom flask and then stirred and heated at 60°C for 2 h under a nitrogen atmosphere. After that, the resulting nanospheres were washed with deionized water three times and twice with methanol, then dried into powders at room temperature under vacuum (Figure 1).

### 2.5. Recommended procedure for sorption and desorption of heavy metal ions

A series of sample solutions containing heavy metal ions were transferred into a 1 L beaker. The pH of the solution was adjusted to 6.0 using 0.01-0.1 mol L^-1 ^HCl and/or NaOH solutions. After that, 0.11 g of the sorbent was added to the solution and the mixtures were dispersed by ultrasonication for 10 min at room temperature to attain equilibrium, and then magnetically separated (Figure 1). Then, the sorbent was washed with deionized water and afterwards, the metal ions retained on sorbent were eluted with the solution of the mixture of 6.0 mL of 1.0 mol L^-1 ^HNO_3_. The analytes in the eluate were then determined by F-AAS.

## 3. Results and discussion

### 3.1. Characteristics of modified magnetite nanoparticles

**Figure 2:**
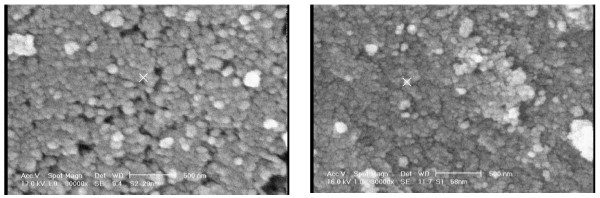
**SEM images of synthesized magnetite nanoparticles (left) and modified magnetite nanoparticles (right)**.

The surface and textural morphology of silica coated magnetite nanoparticles by SEM image is illustrated in Figure 2. As shown in Figure 2, the naked magnetite nanoparticles had a mean diameter of 29 nm. Using the ultrasonic vibrations caused the prepared nanoparticles were smaller and more homogenized particles [30]. After modification process, the modified nanoparticles prepared are in the range of 58-73 nm in diameter. This shows that the magnetite nanoparticles have been completely coated by the silica and SA. Also, this could be attributed to the reaction occurring only on the particle surface, and thus our attempt to prepare SA-silica coated magnetite nanoparticles in this work has been achieved.

**Figure 3:**
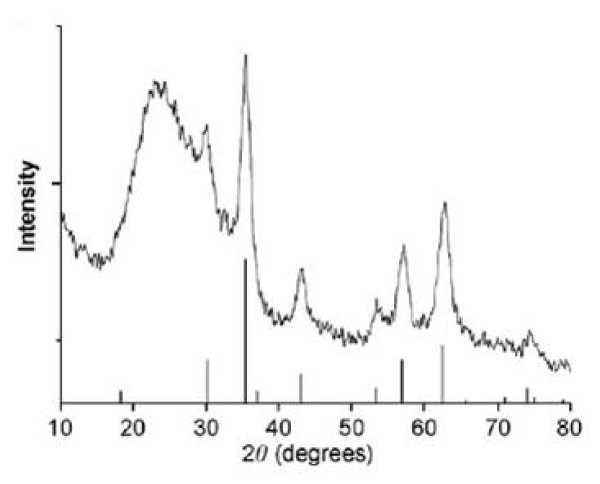
**X-ray diffraction pattern of silica coated magnetite nanoparticles**.

The typical X-ray diffraction (XRD) profile of silica-coated magnetite nanoparticles is shown in Figure 3. The broad peak at around 2θ = 20° in the XRD pattern is due to the amorphous silica shell on the surface of the magnetite nanoparticles. The characteristic peaks of the magnetite nanoparticles were also clearly identified in the XRD pattern for a standard magnetite pattern (Joint Committee on Powder Diffraction Standards (JCPDS) file no. 19-0629) [30,31]. Also, with comparison of peaks of silica-coated magnetite nanoparticles and silica-coated magnetite nanoparticles modified with SA concluded although the magnetic particle surfaces were coated with SA, the very distinguishable FCC peaks of magnetite crystal were observed, which means that these particles have the phase stability. The different functional groups such as hydroxide and carboxylylic did not affect on crystallinity and morphology in this study [32].

The specific surface area was determined using the Brunauer-Emmett-Teller (BET) equation applied to the adsorption data on nitrogen adsorption/desorption experiments. The results of the BET method showed that the average specific surface area of silica-coated magnetite nanoparticles modified with SA was 41.62 m^2 ^g^-1^. It can be concluded from these values that this type adsorbent is nanoparticles with relatively large specific surface area.

**Figure 4:**
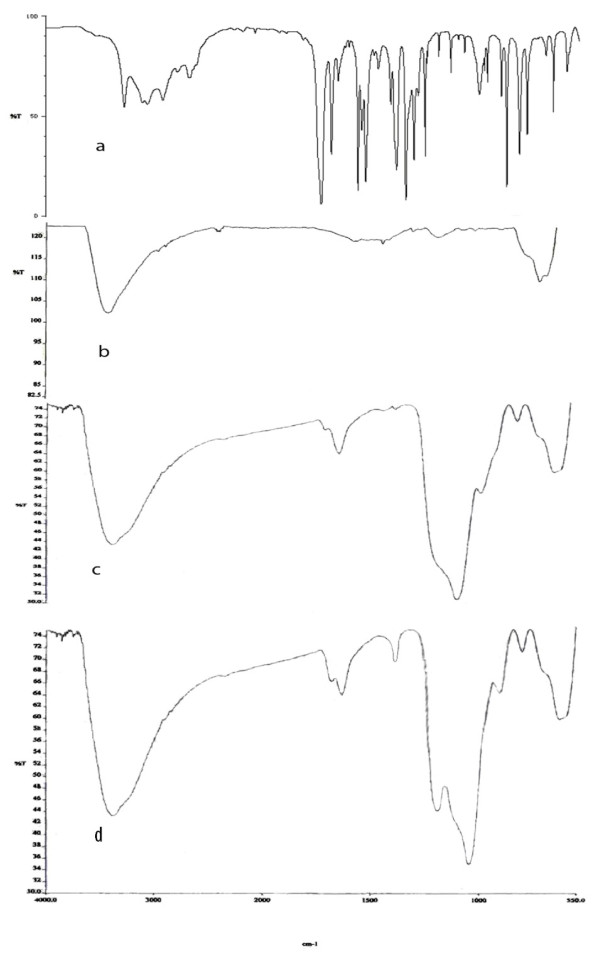
**FT-IR spectra (a) salicylic acid (b) Fe_3_O_4 _nanoparticles (c) SiO_2 _coated Fe_3_O_4 _nanoparticles (d) SiO_2 _coated Fe_3_O_4 _nanoparticles modified with salicylic acid**.

The adsorbent was subsequently characterized by FT-IR spectral data. The FT-IR spectrum of silica-coated magnetite nanoparticles modified with SA has prominent bands at 1680 cm^-1^, 1486 cm^-1^, and 1387 cm^-1 ^due to carboxylate, OH (bending) and phenolic group vibrations, respectively (Figure 4). This supports the immobilizing of SA onto silica-coated magnetite nanoparticles. The red shifts of the two peaks namely hydroxyl and carboxylic by 20-25 cm^-1 ^for metal loaded adsorbent further suggest that chelation with salicylic acid functionality is responsible for the sorption of metal ions by adsorbent. Furthermore, the adsorbent shows good chemical stability with no less of capacity up to 4.0 mol L^-1 ^of HCl/HNO_3_/H_2_SO_4 _used for stripping of metal ions. It can withstand alkaline medium up to 3.0 mol L^-1 ^of NaOH.

### 3.2. Variables Optimization of preconcentration process

#### 3.2.1. Effect of pH for metal ions uptake

**Figure 5:**
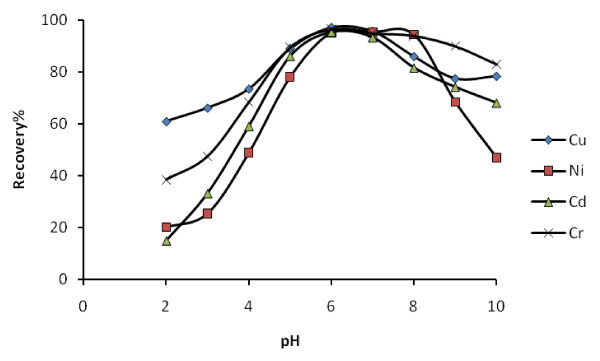
**Relation between pH and recoveries of analytes (N = 3)**.

Solution acidity can show either of two different effects on metal adsorption: protonation of binding sites of the chelating molecules, and complexation or precipitation many metal ions by hydroxide ion. Therefore, since the solution pH is an important parameter to obtain quantitative recovery for heavy metal ions, this was the first parameter that was optimized. The influence of pH on the preconcentration of target metal ions over the pH range from 2.0 to 10.0 was studied (keeping the other parameters constant). As could be seen from Figure 5, quantitative recovery was obtained for Cr(III), Cu(II), Ni(II) and Cd(II) within the pH range 5.0-7.0. This may be attributed to the presence of free lone pair of electrons on oxygen atoms, which are suitable functional sites for coordination with the metal ions. The decrease in recovery at pH values lower than 5.0 may be due to the competition of proton with cations in binding to donor atoms. Considering these facts and in order to avoid an abrupt change in adsorption (which may occur due to minor changes in the pH), and also to preconcentrate of these ions simultaneously, pH 6.0 was selected as the optimal pH for all subsequent experiments.

#### 3.2.2. Effect of contact time

**Figure 6:**
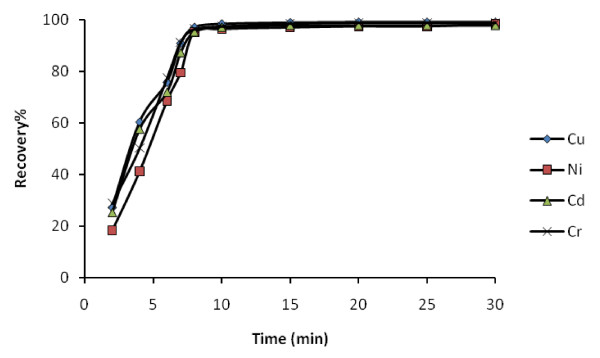
**Effect of contact time on recovery percentage of 20 μg L^-1 ^heavy metal ions; pH 6.0; adsorbent 0.1 g**.

The efficiencies of the analytes deposition depend on the contact time of sample with the solid phase. It is necessary to require the preconcentrate of metal ions in short time. In this regard, replicate sets of analytes and adsorbents were prepared and investigating at different time intervals 2, 5, 8, 10, 15, 20, 30 min. Results showed that the rate of uptake of the analytes was quite high (Figure 6). Adsorption of Cd(II), Cu(II), Ni(II) and Cr(III) from the solution reached more than 95% at about 8 min. Therefore, ultrasonication time of 10 min was selected for further works.

#### 3.2.3. Effect of the adsorbent amount

**Figure 7:**
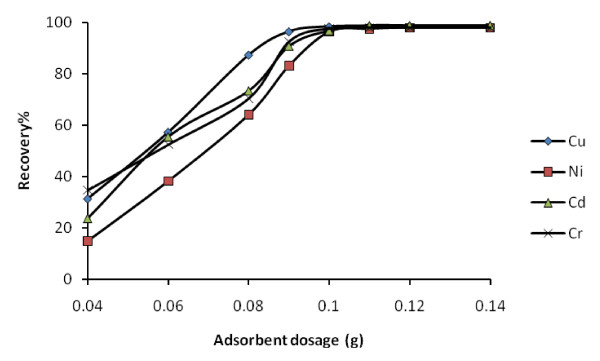
**Recovery percentage of metal ions at different adsorbent dosage**.

The amount of adsorbent is another important parameter to obtain quantitative recovery. For this reason the amounts of silica-coated magnetite nanoparticles modified with SA was optimized. The influence of the adsorbent amount was tested in the range of 0.04-0.14 g. According to the results quantitative recoveries were obtained when nano-sized adsorbent amount was above 0.10 g (Figure 7). Therefore, 0.11 g of adsorbent was used in all experiments.

#### 3.2.4. Effect of sediment time

Conventional SPE usually requires filtration or centrifugation to separate the adsorbent from aqueous solutions, which makes the method time-consuming. In this study, the adsorbent could be separated rapidly from the sample solution using an external magnetic field, due to the superparamagnetism of these nanoparticles. The effect of sediment time on the recovery of metal ions was investigated, and no significant effect was seen when the sedimentation time was greater than 60 s. A sediment time of 1 min was therefore selected in subsequent experiments.

#### 3.2.5. Effect of the type, concentration and volume of the eluent

The selection of suitable eluent was a difficult problem. As could be seen from Figure 4, the uptake of these metal ions was negligible at low pH; therefore, the acidic eluent is the best solution to obtain efficient extraction. The optimal eluent was a difficult problem because of the limitation of FAAS to tolerate organic solvents while the eluent should not destroy the solid phase. Various acids were used to identify the best eluent for the adsorbed metal ions on the adsorbent. The results are given in Table 1. Deionized water was found to be unsuitable for the purpose of elution as <0.5% recovery was achieved indicating that the metal ions were retained by the adsorbent by some strong bonding forces. Among of different eluents used, 1.0 mol L^-1 ^of HNO_3 _provided higher recovery and reproducibility. Therefore, this solution was chosen as an eluent for the metal ions from nano-sized adsorbent.

**Table 1:** Effect of type and concentration eluting solution (4 mL) on analytes recovery (%)

Eluent		Recovery% (N = 5)^a^			
**Type**	**Concentration (mol L^-1^)**	**Cu(II)**	**Ni(II)**	**Cd(II)**	**Cr(III)**

HCl	1.0	22 ± 2	33 ± 2	29 ± 2	15 ± 2
	2.0	25 ± 1	70 ± 1	42 ± 1	29 ± 1
	4.0	90 ± 3	92 ± 2	85 ± 2	31 ± 2
H_2_SO_4_	1.0	19 ± 1	25 ± 2	34 ± 1	32 ± 1
	2.0	26 ± 3	32 ± 2	40 ± 1	36 ± 3
	4.0	54 ± 2	47 ± 1	51 ± 2	43 ± 2
HNO_3_	1.0	98 ± 1	97 ± 1	99 ± 1	98 ± 1
	2.0	98 ± 1	97 ± 1	99 ± 1	97 ± 1
	4.0	96 ± 2	97 ± 2	98 ± 2	98 ± 2
	1.0	36 ± 2	28 ± 2	20 ± 2	24 ± 2
HClO_4_	2.0	41 ± 1	28 ± 2	26 ± 1	26 ± 2
	3.0	45 ± 2	28 ± 1	35 ± 2	26 ± 2
CH_3_COOH	1.0	9 ± 3	18 ± 2	9 ± 3	8 ± 2
	2.0	12 ± 2	19 ± 2	11 ± 2	14 ± 2
	4.0	13 ± 2	18 ± 1	12 ± 2	16 ± 2

^a ^Mean of five determinations ± standard deviation

Subsequent experiments showed that even 2.0 mL of the eluent solution was enough for elution of metal ions, however, in all experiments metal ions were eluted by 4.0 mL of 1.0 mol L^-1 ^of HNO_3 _solution because this volume was necessary for reading absorption signal of analytes by F-AAS.

#### 3.2.6. Effect of the sample volume

Due to the low concentrations of trace metals in real samples, these analytes should be taken into smaller volumes for high preconcentration factor by using sample solutions with large volumes. Therefore the maximum applicable sample volume was determined by increasing the dilution of metal ion solution, while keeping the total amount of loaded metal ion fixed for analytes. Different feed volumes varied between 50 and 1000 mL. The recoveries were found to be stable until 800 mL and were chosen as the largest sample volume to work. In this study, the final solution volume to be measured by F-AAS was 4.0 mL; therefore the preconcentration factors were 200 for all metal ions. At volumes higher than 800 mL probably the analyte ions are not sorbed effectively because of low amount of adsorbent in those volumes. As stated previously, the final solution volume, after eluting the metal ions, was 4.0 mL, therefore the preconcentration factors of 200 was obtained for all analytes.

### 3.3. Stability and reusability of adsorbent

The reusability and stability of the adsorbent was investigated. The capacity of the modified adsorbent was found to be apparently constant (less than 3%) after the repeated use of more than 9 cycles of sorption and desorption of the target analytes.

### 3.4. Effect of potentially interfering ions

In view of the fact that flame atomic absorption spectrometry provides high selectivity, the only interference may be attributed to the preconcentration step. For application of recommended solid phase extraction to real samples, effects of some interfering species on the recovery of metal ions were investigated with the optimized procedure. Various species, which are inevitably associated with heavy metals, may interfere in the final determination through precipitate formation, redox reactions or competing complexation reactions. In order to assess the analytical applicability of the adsorbent to real samples, common chemical species such as sodium citrate, sodium tartrate, sodium oxalate, humic acid, fulvic acid, NO_3_^-^, CO_3_^2-^, NH_4_^+^, SO_4_^2-^, PO_4_^3-^, Cl^-^, K^+ ^and Na^+ ^were checked for any interference in the sorption of these metals. The tolerance limit is defined here as the species concentration causing a relative error smaller than ± 5% related to the preconcentration and determination of the analytes. The tolerable limits of interfering ions are given in Table 2.

**Table 2:** Effects of the matrix ions on the recoveries of the examined metal ions.

	Potentially interfering ion/analyte fold ratio				
		
Added salt	Potentiall interfering species	Cu(II)	Ni(II)	Cd(II)	Cr(III)
NaCl	Na^+^,	12000	12000	12000	10000
KCl	K^+^,	12000	12000	12000	10000
NaCl	Cl^-^	12000	12000	12000	10000
Na_2_SO_4_	SO_4_^2-^	12000	12000	12000	10000
NaNO_3_	NO_3_^-^	12000	12000	12000	10000
C_6_Na_3_H_5_O_7_.2H_2_O	C_6_H_5_O_7_^3-^	12000	12000	12000	10000
C_4_Na_2_H_4_O_6_.2H_2_O	C_4_H_4_O_6_^2-^	12000	12000	12000	10000
Na_2_C_2_O_4_	C_2_O_4_^2-^	12000	12000	12000	10000
Na_2_CO_3_	CO_3_^2-^	12000	12000	12000	10000
Ca_3_(PO_4_)_2_	PO_4_^3-^	12000	10000	10000	10000
NH_4_Cl	NH_4_^+^	500	400	300	300
Ca_3_(PO_4_)_2_	Ca^2+^	500	400	300	300
BaCl_2_	Ba^2+^	12000	10000	10000	10000
MgCl_2_	Mg^2+^	12000	10000	10000	10000
Al(NO_3_)_3_	Al^3+^	12000	12000	12000	12000
Mn(NO_3_)_2_	Mn^2+^	12000	12000	12000	12000
Co(NO_3_)_2_	Co^2+^	100	70	100	100
Zn(NO_3_)_2_	Zn^2+^	100	70	100	100
FeCl_3_	Fe^3+^	80	70	25*	50
Humic acid	Humic acid	50	65	70	60
Fulvic acid	Fulvic acid	55	50	45	45

* Mask with F^-^.

The allowable amount of Fe(III) ions as an interfering species was lower than the other investigated species in preconcentration of cadmium. The usage of masking agent such as NH_4_F for this interfering species in the present method resulted in suppressing the effect of Fe(III) interference and improving the selectivity. However, the tolerance ratio for Fe(III) ion could be raised to 700 times when 2 mL of 0.25 mol L^-1 ^NH_4_F solution is also added when necessary.

### 3.5. Adsorption capacities

The capacity of the adsorbent is an important factor because it determines how much adsorbent is required to quantitatively remove a specific amount of metal ions from the solutions. The adsorption capacity was tested following the batch procedure. 110 mg of sorbent was equilibrated with 800 mL of various concentrations of Cu(II), Ni(II), Cd(II) and Cr(III) for 1 h. In order to reach the "saturation", the initial metal ions, concentrations were increased till the plateau values (adsorption capacity values) obtained. The results showed that adsorption capacity of various metal ions probably differ due to their size, degree of hydration and the value of their binding constant with the adsorbent. The maximum adsorption capacity has been found to be 39.9, 39.8, 27.8 and 17.3 mg g^-1 ^for Cu(II), Cr(III), Cd(II) and Ni(II), respectively.

### 3.6. Analytical precision and detection limits

Under the selected conditions, eight portions of standard solutions were enriched and analyzed simultaneously following the general procedure. Linearity was maintained between 0.73 μg L^-1 ^to 0.40 mg L^-1 ^for copper; 0.91 μg L^-1 ^to 0.35 mg L^-1 ^for nickel; 0.44 μg L^-1 ^to 0.64 mg L^-1 ^for chromium and 0.37 μg L^-1 ^to 0.45 mg L^-1 ^for cadmium, in initial solution. The detection limit (DL) of the present work was calculated under optimal conditions after the application of preconcentration and separation procedure of blank solutions analyzed by F-AAS. The DL was calculated as DL = *kS*_b_/*m*, where *k *is equal to 3 according to the desired confidence level (95%), *S*_b _is the standard deviation of the blank signal and *m *is the slope of the analytical curve (n = 8). The detection limits were found to be 0.15, 0.22, 0.27 and 0.11 μg L^-1 ^for Cr(III), Cu(II), Ni(II) and Cd(II), respectively. The relative standard deviation (RSD) of the eight replicate determinations was lower than 4.0% (Cr(III): 3.1%; Cu(II): 2.2%; Ni(II): 3.3%; Cd(II): 2.6%), which indicated that the method had good precision for the analysis of trace target ions in solution samples.

### 3.7. Application to real samples

Certified reference materials were analyzed by developed method. The results are given in Table 3. The results show that the results are good agreement with the certified values for the investigated analyte ions. The proposed method was applied to a various water samples. The results were given Table 4. Proposed solid phase extraction method for determination of these metals in some water samples were applied successfully. Recovery values can be quantitatively except in all samples. Multivitamin capsule, IMS and hydrogenated oil samples were investigated as samples with complex matrices. These results indicate the applicability of the developed procedure; for selective preconcentration of target analytes; and that it is free of interference (Table 5). Also, the proposed procedure has been applied to the determination of copper, nickel, chromium and cadmium content; in tomato and spinach samples. The results are given in Table 6. As can be seen from the results in Table 6 the metal ions were quantitatively recovered from the food samples; by the proposed procedure. These results demonstrate, the applicability of the procedure for target ions determination in water samples.

**Table 3:** Results for metal ions determination in certified reference samples obtained using the optimum conditions.

Samples	Certified value (μg g^-1^)	Found by proposed method (μg g^-1^)^a^	Calculated value of t-test^b^
Vehicle exhaust particulates (NIES-8)	Cd:1.1, Cr:3.3, Cu:67, Ni:18.5	Cd: 1.06 ± 0.04, Cr: 3.2 ± 0.2; Cu: 66.5 ± 0.4, Ni: 18.4 ± 0.3	1.96, 2.49, 2.54, 2.74
Human hair (NIES-5)	Cd: 5.2, Cu: 16.3, Ni: 1.8	Cd: 5.0 ± 4.0, Cu: 15.9 ± 2.8, Ni: 1.7 ± 4.8	2.23, 2.44, 2.00, 2.74
Tea leaves (NIES 7)	Cd: 0.030, Cr: 1.3, Cu: 7.0, Ni: 6.5	Cd: 0.028 ± 0.001, Cr: 1.2 ± 0.1, Cu: 6.7 ± 0.1, Ni 6.2 ± 0.1	1.70, 2.60, 2.22, 2.51
Pepperbush (NIES-1)	Ni: 8.7, Cd: 6.7	Ni: 8.8 ± 0.1, Cd: 6.6 ± 0.2,	1.34, 1.83
Zinc base diecasting alloy C (NBS-627)	Cu: 1320, Cd:51, Ni:29	Cu: 1310.2 ± 1.2, Cd: 49.1 ± 4.1, Ni: 27.5 ± 4.6	1.39, 2.11, 2.65

(*N *= 5)

^a ^Mean of five determinations ± standard deviation

^b ^t_exp _shows the experimental student-t values at confidence limit 95%.

**Table 4:** Results for metal ions determination in various water samples obtained using the optimum conditions.

Sample	Added (μg L^-1^)	Found (μg L^-1^) ^a^			
		
		Cu(II)	Ni(II)	Cd(II)	Cr(III)
Tap water	0.00 10.00	1.95 ± 0.45 12.12 ± 0.12	5.82 ± 0.31 15.98 ± 0.35	ND^b ^10.03 ± 0.07	ND 10.12 ± 0.09
Canal water	0.00 10.00	4.93 ± 0.15 14.87 ± 0.11	6.48 ± 0.36 16.32 ± 0.38	0.83 ± 0.09 10.68 ± 0.12	5.85 ± 0.38 16.17 ± 0.29
Sewage water	0.00 10.00	6.93 ± 0.16 17.19 ± 0.12	69.88 ± 0.41 80.09 ± 0.37	4.65 ± 0.13 14.91 ± 0.21	52.75 ± 0.26 63.12 ± 0.32
River water	0.00 10.00	3.64 ± 0.13 13.48 ± 0.22	8.92 ± 0.61 19.13 ± 0.52	1.67 ± 0.05 11.95 ± 0.24	1.32 ± 0.25 11.44 ± 0.42

(*N *= 5)

^a ^Mean of five determinations ± standard deviation

^b ^Not detected

**Table 5:** Results for metal ions determination in various samples obtained using the optimum conditions.

Samples	Reported value (μg g^-1^)	Found by proposed method (μgg^-1^)^a^
Multivitamin	Cu: 3.98	Cu:3.95 ± 0.13
Infant milk substitute	Cu:2.90	Cu: 2.89 ± 0.25
Hydrogenated oil	Ni: 4.50	Ni:4.3 ± 0.3

(*N *= 5)

^a ^Mean of five determinations ± standard deviation

**Table 6:** Results for metal ions determination in food samples obtained using the optimum conditions.

Sample	Added (μg g^-1^)	Found (μg g^-1^) ^a^			
		
		Cu(II)	Ni(II)	Cd(II)	Cr(III)
Tomato	0.00 10.00	2.56 ± 0.55 12.72 ± 0.37	8.95 ± 0.24 19.24 ± 0.35	1.12 ± 0.05 11.26 ± 0.04	3.72 ± 0.40 13.49 ± 0.09
Spinach	0.00 10.00	0.56 ± 0.04 10.69 ± 0.15	1.53 ± 0.12 11.29 ± 0.18	0.08 ± 0.02 10.05 ± 0.02	5.85 ± 0.38 16.17 ± 0.29

(*N *= 5)

^a ^Mean of five determinations ± standard deviation

## 4. Conclusion

A simple, sensitive and selective method was developed for the preconcentration of cadmium, copper, nickel and chromium in various real samples. In summery, silica coated Fe_3_O_4 _nanoparticles modified with SA with well defined diameter prepared by such a simple, time-saving and low cost route using sol-gel method combined ultrasonic stirring. These nanoparticles have relatively high adsorption as compared to the similar materials because of their smaller size. The size of the produced modified maghemite nanoparticles was determined by X-ray diffraction (XRD) analysis and scanning electron microscopy (SEM). The present method has following advantages over reported methods. Synthesized adsorbent is distinct in terms of sensitivity, selectivity towards investigated metal ions. Also, these magnetic nanoparticles carrying the target metals could be easily separated from the aqueous solution simply by applying an external magnetic field; no filtration or centrifugation was necessary. Furthermore, the proposed method gives an efficient and cost effective method with very low detection limits and good relative standard deviation and can be applied to the determination of traces of these ions in various real samples.

## Competing interests

The authors declare that they have no competing interests.

## Authors' contributions

AA carried out the survey of prepared adsorbent, participated in the design of the study. HB carried out the synthesis of adsorbent and performed the some of experimental sections. MRS performed the experimental sections, participated in the sequence alignment and drafted the manuscript. All authors read and approved the final manuscript.
